# Improved Classification of *Orthosiphon stamineus* by Data Fusion of Electronic Nose and Tongue Sensors

**DOI:** 10.3390/s101008782

**Published:** 2010-09-28

**Authors:** Ammar Zakaria, Ali Yeon Md. Shakaff, Abdul Hamid Adom, Mohd Noor Ahmad, Maz Jamilah Masnan, Abdul Hallis Abdul Aziz, Nazifah Ahmad Fikri, Abu Hassan Abdullah, Latifah Munirah Kamarudin

**Affiliations:** Sensor Technology and Applications Group (STAG), Universiti Malaysia Perlis (UniMAP), 01000, Kangar, Perlis, Malaysia; E-Mails: aliyeon@unimap.edu.my (A.Y.M.S.); abdhamid@unimap.edu.my (A.H.A.); mohdnoor@unimap.edu.my (M.N.A.); mazjamilah@unimap.edu.my (M.J.M.); abdulhallis@unimap.edu.my (A.H.A.Z.); naffe_five@yahoo.com (N.A.F.); abuhassan@unimap.edu.my (A.H.A.); munirahkamarudin@gmail.com (L.M.K.)

**Keywords:** electronic nose, electronic tongue, data fusion, PCA, LDA, *Orthosiphon stamineus*

## Abstract

An improved classification of *Orthosiphon stamineus* using a data fusion technique is presented. Five different commercial sources along with freshly prepared samples were discriminated using an electronic nose (e-nose) and an electronic tongue (e-tongue). Samples from the different commercial brands were evaluated by the e-tongue and then followed by the e-nose. Applying Principal Component Analysis (PCA) separately on the respective e-tongue and e-nose data, only five distinct groups were projected. However, by employing a low level data fusion technique, six distinct groupings were achieved. Hence, this technique can enhance the ability of PCA to analyze the complex samples of *Orthosiphon stamineus*. Linear Discriminant Analysis (LDA) was then used to further validate and classify the samples. It was found that the LDA performance was also improved when the responses from the e-nose and e-tongue were fused together.

## 1. Introduction

At present, *Orthosiphon stamineus* is one of the herbs being commercialized for pharmaceutical purposes in Malaysia with most potential. It is said to be rich in health-related benefits for many ailments and is normally consumed in the form of a herbal tea. Locally, the commercial potential of *Orthosiphon stamineus* has become very attractive because it is easy to cultivate under tropical climate conditions. However, at this moment, there is no rapid technique to assess and evaluate the quality of commercial *Orthosiphon stamineus* products.

In the case of teas, the quality classification of black tea, green tea, and oolong tea are performed using organoleptic methods. Tea samples are prepared according to certain procedures and later tested by human panels. Likewise, *Orthosiphon stamineus* tea quality assessment should follow the same approach. However, human panels have many disadvantages such as being prone to fatigue and inconsistencies due to overwhelming flavours and aromas in the samples. Due to that reason, many researchers have performed e-nose assessments on brewed *Orthosiphon stamineus* tea to determine the ‘aroma concentration’, quality, etc. Unfortunately, discrimination of herbal beverages by electronic noses (e-noses) has been known to be physically challenging due to the influence of water vapour and temperature drift [1]. Some researchers [2,3], have suggested the use of pre-concentrators to reduce this effect, while [4] performed baseline manipulation to overcome the problem. However, no attempt has been reported to confirm the effect of water vapour by comparing the aroma of dried and brewed *Orthosiphon stamineus*. Thus, in this paper, the headspace of dried leaves and *Orthosiphon stamineus* tea infusions were measured, compared and further analyzed. This procedure is essential to obtain the right experimental setup and sniffing parameters.

In recent years, there have been a number of reported works on the assessments of agriculture-based products using the e-nose and e-tongue, but separately [5–8]. A number of analysis techniques have such as multivariate analysis, neural networks and many more that were focused on qualitative and quantitative analysis been developed and applied to the data from these sensors.

Despite of these techniques and methods, the e-nose can only evaluate volatile compounds or the aroma of a liquid in the headspace (*i.e.*, evaluating the strength of the aroma concentration), while an e-tongue can discriminate the concentration of active compounds in a complex solution [9–11]. For example, [5–8] have reported that single modality techniques were able to discriminate various agro-based products according to the produce quality, different geographical origin, farming practices and postharvest processing. However, these evaluations may be influenced by other factors such as temperature drifts, changes in humidity, and not solely on the ability of these single-modal systems. This is because the reported work does not provide conclusive justifications.

The limitations of an e-nose to evaluate active compounds in *Orthosiphon stamineus* tea products lead to the idea of introducing a fusion technique by combining both the e-tongue and e-nose. This combination should produce different responses and may provide further information. This is somewhat similar to the human sensory system, whereby the smell and taste sensation interact very closely with each other [12,13]. Thus, data fusion technique of different modalities [1,14,15] could provide better information compared to single modal systems [16–18].

This paper presents an investigation which combines information from the two modalities to evaluate and discriminate commercial *Orthosiphon stamineus* tea product samples. This technique can in fact be a fast approximation of analyses performed using the costly and more elaborate High-Performance Liquid Chromatography (HPLC) or Gas Chromatography-Mass Spectrometry (GCMS).

## 2. Materials and Methods

### 2.1. Sample Selection and Preparation

In this experiment, six samples each were taken from five different brands. These 30 samples were obtained from commercial sources in different batches, while another six samples were fresh coarsely ground dried leaves obtained from home-grown plants (UniMAP’s Sungai Chuchuh plantation) and used as control samples (labeled as Agro). Each sample contained 2 g of dried *Orthosiphon stamineus* tea. In total, 36 samples of dried *Orthosiphon stamineus* tea from six different sources prepared for the experiment.

For the assessment of dried leaves, each sample were filled up in a vial and sealed. The vials were purged with helium before being filled with ground dried leaves. The vials were then kept for 10 minutes until the headspace of the vials equilibrated. The measurements were performed under room temperature at 26 °C.

For the *Orthosiphon stamineus* tea infusions, 200 mL boiled distilled water was used to prepare each tea sample. It was brewed for three minutes, filtered and left to cool down to 40 °C before e-tongue measurements were taken. The aromas of those infusions were evaluated immediately after the e-tongue measurement was completed. A summary of the samples used in this experiment, together with the number of replicated measurements by the e-nose (for dried leaves and tea infusion assessments) and e-tongue is shown in Table 1. The colour of the tea infusions was recorded based on visual observations.

### 2.2. Electronic Tongue Setup and Measurement

The e-tongue, using chalcogenide-based potentiometric sensors, comprise seven distinct ion-selective sensors from SENSOR SYSTEM, LLC [19]. These potentiometric sensors were designed to be partially selective. The combination of these sensors as an array will introduce a cross sensitivity effect, which may allow the qualitative and quantitative assessments of complex solutions [10]. Vlasov [10] and Toko [11] have demonstrated that such sensor arrays, together with suitable pattern recognition (PARC) algorithms can mimic the human tongue. Table 2 describes the potentiometric sensors used in this experiment. This e-tongue system is implemented by arranging an array of potentiometric sensors around the reference probe. Each sensor output was connected to the analogue input of a data acquisition board (NI USB-6008) from National Instruments [20] and the reference probe is connected to the common ground of the board.

The sensor array was dipped for two minutes in 10% ethanol concentration (stirred at 400 rpm) at the beginning of the experiment and later rinsed with distilled water for two minutes. After each sampling, the sensor array was rinse twice using distilled water (stirred at 400 rpm for two minutes) to remove any residues from previous sample sticking on the e-tongue and contaminating the next sample. In each measurement, the sensor array was steeped simultaneously (sensor tip 2 cm below the solution level) and left for five minutes, and the potential readings were recorded for the whole duration.

### 2.3. Electronic Nose Setup and Measurement

The e-nose employed is the Cyranose320 from Smith Detection™ and has 32 non-selective individual sensors made up of different types of polymer matrix, blended with carbon black and arranged as an array. The same principle explained above for the e-tongue is adopted by the e-nose to discriminate complex odours. Preliminary experiments were performed to determine the optimal e-nose measurement parameters. Fifteen seconds baseline purge with 20 s sample draw produced an optimal result (result is not shown). Although there are no exact guidelines on this setting, a general assumption on the sensitivity and sensors response can still be applied in this case. Longer baseline purge was required to ensure residual gases were properly removed, and pump setting was set to the lowest speed during sample draw to enhance and maximize the sensor response. Charcoal filter was used to remove organic volatiles in the ambient.

**Figure 1.**
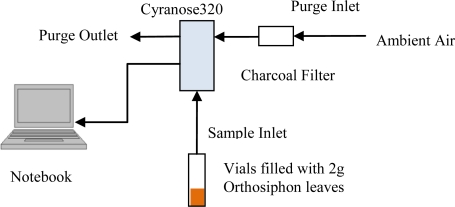
E-nose setup for headspace evaluation of dried *Orthosiphon stamineus* tea.

**Figure 2.**
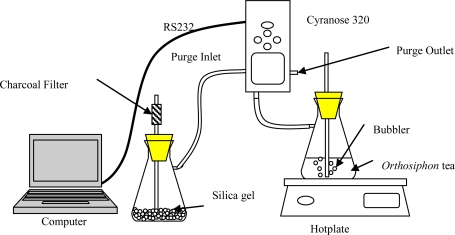
E-nose setup for headspace evaluation of *Orthosiphon stamineus* tea infusions.

The first experiment was carried out using e-nose on dried samples of *Orthosiphon stamineus* tea as shown in Figure 1. The measurement parameters for the e-nose are given in Table 3. The e-nose setup for the *Orthosiphon stamineus* tea infusions is illustrated in Figure 2 and the setting on the sniffing cycle is also indicated in Table 3. Silica gel with charcoal filter was used in this setup to provide dry and clean air for baseline recovery process. The purging duration was set to 80 seconds. This setup is important to reduce the effect of accumulated water vapour inside the sensor chamber. The temperature of tea infusions was controlled at 40 °C during the headspace measurement.

### 2.4. Data Analysis

Before the analysis, the fractional measurement method was applied to pre-process the data for both modalities. This is often known as baseline manipulation. The baseline (initial value) is subtracted and then divided by the sensor response. The result is a dimensionless and normalized *S_frac_*, where:

(Equation 1) $$S_{\mathrm{frac}}=[S_{\text{max}}-S_{0}]/S_{0}$$

This gives a unit response for each sensor array output with respect to the baseline, which compensates for sensors that have intrinsically large varying response levels [4]. It can also further minimize the effect of temperature and humidity drifts. For the e-tongue measurements, S_0_ (baseline reading) is the reading of distilled water, while S_max_ is the sensor readings when steeped in the *Orthosiphon stamineus* tea infusions samples. The steeping cycle was repeated three times for each tea sample and the average was obtained.

In the case of the e-nose, S_0_ was taken during the baseline purge with ambient air and S_max_ was performed during the sample draw. Each sampling cycle was repeated 10 times and the average was obtained for each of the six tea samples from different teas. The procedure was performed on both e-nose assessments of dried leaves and tea infusions.

After the above operation, the data *S_frac_* was further scaled to zero mean and one standard deviation. This is to ensure that all sensor responses were standardized and no particular sensor dominates the result. The data from different modality were process separately and all sensors were used in this analysis. However, instead of looking at a specific sensor, the multiple sensors’ responses would give more meaningful information [15]. The unsupervised exploratory data analysis technique such as Principal Component Analysis (PCA) was identified as a suitable method to visualize patterns in the data, especially since the sensors are correlated [21,22].

Each individual modality was projected separately by PCA based on correlation matrix. An adequate number of dimensions projected by PCA were determined based on principal components (PCs) that have achieved cumulative variance of 80% or more. The same method is applied for the e-tongue data.

Further analyses to validate and classify those six different classes were performed using Linear Discriminant Analysis (LDA). It was done separately on each modality, as well as on the fused e-nose and e-tongue data. The LDA is a supervised pattern recognition method and is based on the determination of linear discriminant functions of which inter-group variance is maximized and within-group variance is minimized [15]. The PCA and LDA were computed using MATLAB 7.0 and SPSS Statistics 17.0, respectively.

### 2.5. Data Fusion

Low level fusion is performed by combining the information provided by different sensors in different modalities. There are many methods to perform this fusion *i.e.*, using neural network, template methods, and cluster algorithms [14–16]. In this experiment, PCA and LDA were chosen to perform the low level fusion. The requirement for this method is that the sensors for both modalities must commensurate [23,24].

PCA was used to analyze the behavior or the grouping of the data [21]. Further training, validation and classification between sample groups of the data fusion were performed using LDA. Cross-validation using leave-one-out method was carried out and variable selection was accomplished using Wilks’ lambda test. Fisher linear discriminant function was also applied in this analysis.

The electronic nose data consists of 36 data samples with 32 variables from the sensor response. Data from the tea infusion assessment was selected for the fusion process. It was selected based on the total percentage of variance accumulated in the first two principal components (PCs). The e-tongue data consists of 36 data samples with seven variables from the sensor response. Hence, the combined dataset from the e-nose and e-tongue consists of 36 data samples with 39 variables. To ensure these dataset are standardized, this new dataset (after being combined) was scaled before performing the PCA and LDA.

## 3. Results and Discussion

### 3.1. Principal Component Analysis (PCA)

Each modality (e-nose data and e-tongue data) was processed separately. Prior to PCA projection, a number of adequate PCs were determined. The amounts of variance (%) of the first five principal components for three different experiments are shown in Table 4. The amount of accumulated variance in the first two principal components accounted for more than 80% and this suggests that only the first 2 PCs should be considered or adequate enough for further PCA analysis.

**Figure 3.**
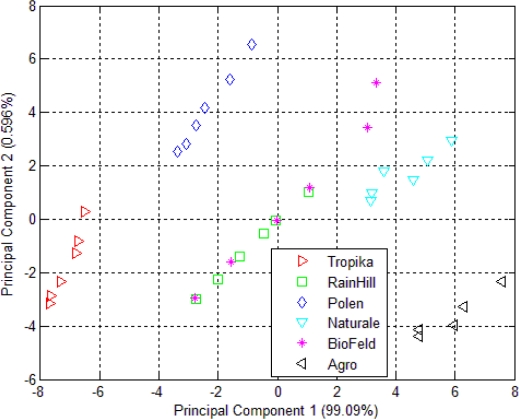
PCA plot of 32 e-nose sensors responses for dried *Orthosiphon stamineus*.

The aroma of the dried *Orthosiphon stamineus* from six different sources was measured using an e-nose and projected using a PCA plot as shown in Figure 3. Although the data samples were clustered into separate groups, the clustering within each group is fairly wide. This implies that the headspace of 2 g of dried *Orthosiphon stamineus* does not produce enough volatiles to excite the sensors. The plot also indicates a close resemblance between samples from the Rainhill and Biofeld brands, where both are clustered as one group. The rest of the samples can be clustered into several other distinct groups.

**Figure 4.**
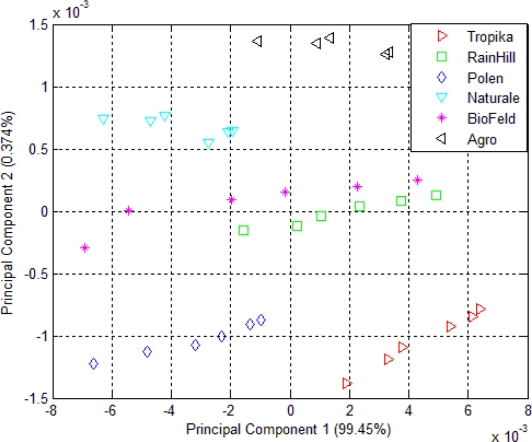
PCA plot of 32 e-nose sensors responses for *Orthosiphon stamineus* infusions.

The result of the clustering using PCA of the headspace measurements of *Orthosiphon stamineus* infusions is shown in Figure 4. The clustering behavior is similar to that shown in Figure 3. Infusions made from samples by the RainHill and BioFeld brands were once again clustered close together and may be perceived as one group in the projection plot. Those made from samples by Agro, Polen, Tropika, Naturale and Terinai brands were clearly separated from each other. The scales of both axes were very small compared to Figure 3. The separations between samples were also insignificant. It is possible to some extent, that water vapour affects the measurements. This is one of the common drawbacks in conducting polymer based sensor where it is prone to be affected by humidity changes unlike human nose that can adapt to these conditions.

Although the positions of each cluster for the dried and brewed samples shown in Figures 3 and 4 are different, the grouping behavior is still similar. Hence, it can be assumed that the effect of water vapour on the sensors’ responses due to the use of the bubbler method in this experiment is minimal and can be ignored. In another word, dried or infusions of the different brands can still be discriminated by the e-nose. Therefore, the responses of the 32 sensors of the e-nose can be used for the next stage of the experiment for the data fusion technique.

**Figure 5.**
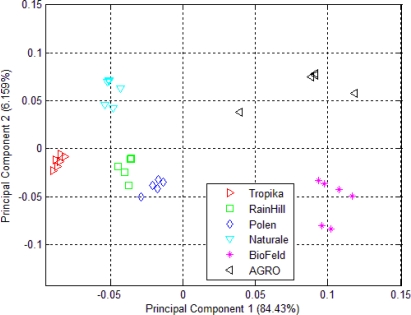
PCA plot of seven e-tongue sensors responses for *Orthosiphon stamineus* infusions.

The PCA plot of six different kinds of *Orthosiphon stamineus* tea using the e-tongue is shown in Figure 5. The results of the e-tongue sensors show a different clustering of the infusions made from the samples of different sources. Infusions from the RainHill and Polen brands are clustered closely and can be assumed as one group. The rest of the groups show good separation. It can also be seen that most of the distance within the individual groups is lower than in the case of the PCA plot of e-nose measurement as shown in Figure 3. On the other hand, the results of e-tongue measurements show a better discrimination than the e-nose.

**Figure 6.**
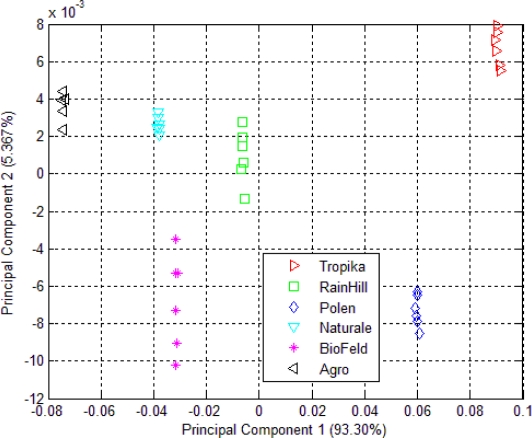
PCA plot using data fusion technique.

Figure 6 shows the results of the data fusion with 39 variables from the e-nose and e-tongue data. Six distinct classes were shown in this PCA plot. It can be observed that the classification performance was greatly improved when both data from the e-nose and e-tongue were combined and complemented each other. These classes are well separated and show significant improvement in confidence level and consistency.

Based on the PCA of the fusion method as shown in Figure 6, the Agro cluster shows small within-group variance. This control samples were freshly prepared and underwent a consistent agricultural practices and postharvest processing. Naturale, Polen and Tropika also exhibit similar patterns. Hence, this could imply that the variation between batches were minimal and possibly due to consistent agricultural practices. However, for BioFeld and RainHill samples, the data are spread widely along the PC2-axis. This again could be due to inconsistencies in their agricultural practices.

### 3.2. Linear Discriminant Analysis (LDA)

**Figure 7.**
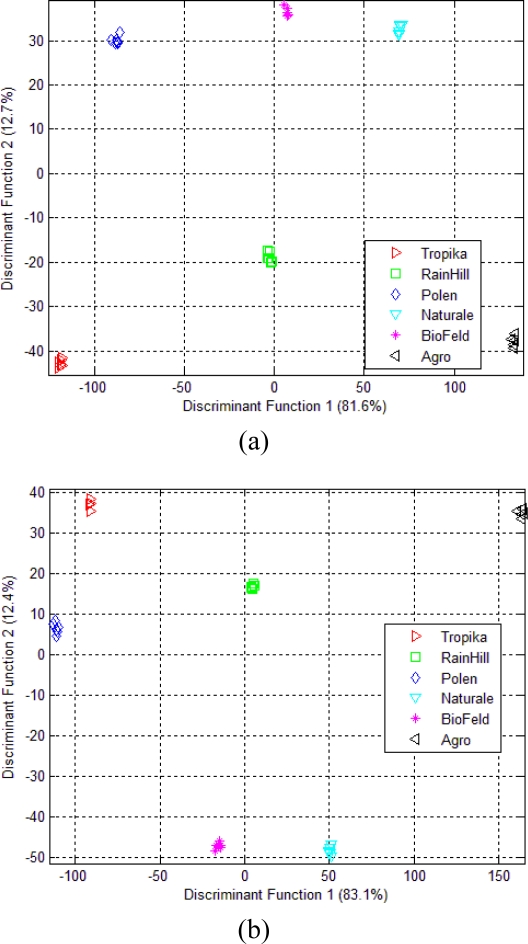
(a) LDA plot for e-nose measurement of dried *Orthosiphon stamineus*; (b) LDA plot using e-nose measurement on *Orthosiphon stamineus* infusions.

For the e-nose data of dried *Orthosiphon stamineus*, LDA is able to classify six different groups, as shown in Figure 7a. The grouping pattern is improved compared to the PCA projection in Figure 4. The separations between the groups are also higher and LDA is able to give 100% correct classification of the different brands. Discriminant Function (DF)1 and DF2 describe 81.6% and 12.7% of the total variance between groups, respectively.

For the case of *Orthosiphon stamineus* infusion, the percentage of total variance in both DF1 and DF2 has now increased to 83.1% and 12.4% respectively, as shown in Figure 7b. Other than that, the pattern of the six different grouping is almost similar. Also, all sensors have strongly contributed to the classification of these six different brands, as shown by the Wilks’ Lambda results in Table 5.

**Figure 8.**
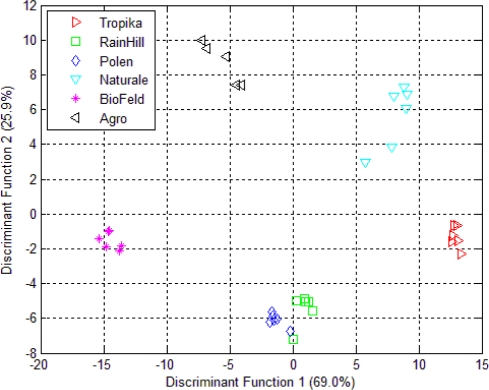
LDA plot using e-tongue measurement on *Orthosiphon stamineus* tea infusions.

The LDA projection for the e-tongue data is shown in Figure 8. The pattern closely resembles the PCA plot in Figure 5. The infusions from the RainHill and Polen brands are clustered closely together and can be assumed as one group. During the validation process, 92.7% of the 36 samples from six different sources were correctly classified. The Wilks’ lambda in Table 5 shows that all the variables contributed towards the classification except sensor 5 and sensor 7 (with values above 0.8), which may be removed from the models.

**Figure 9.**
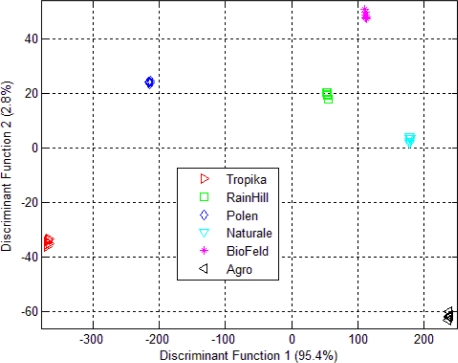
LDA plot using data fusion technique (based on tea infusion assessment of e-nose and e-tongue).

The LDA of the fused data is shown in Figure 9. Again, the e-nose measurement for the *Orthosiphon stamineus* infusions was selected since it has higher total percentage of variance in both DF1 and DF2 compared to the dried samples. The result shows that the LDA is able to give 100% correct classification of different group. The amount of variance for DF1 and DF2 were also improved using this fusion method. The separation between the groups is also increased. The result of Wilks’ lambda test on the data fusion shows that all 39 sensors ‘interact with each other’ and contribute to the classification of the six different groups (shown in Table 5). All 39 sensors can be seen to have significant contribution (values below 0.8 for Wilks’ lamda). In the previous case (*i.e.*, separate modality), sensors 5 and 7 of the e-tongue were found to be insignificant (as mentioned earlier). Hence, the effectiveness of combining the 32 sensors from the e-nose and the 7 e-tongue sensors can be clearly seen.

The performance of LDA to classify the six types is significantly improved using low level data fusion. Also, the success of this method to discriminate and classify all five commercial brands and the control sample shows that there are differences between the samples.

## 4. Conclusions

The results have shown that the two types of *Orthosiphon stamineus* sample preparations (dried and infusions) demonstrated similar data clustering using PCA. However, the dried samples do not produce significant sensor response compared to the infusions. Individual assessments using the e-nose provide a weak classification and discrimination between six different samples and similar result was observed with the e-tongue.

The experiments conducted on the *Orthosiphon stamineus* samples have shown that discrimination using PCA can be improved by applying data fusion. This technique can therefore extend the ability of e-nose and e-tongue when used together to evaluate and classify complex samples. Using PCA, the e-nose was able to discriminate only five out of six different classes. Similar response was observed by the e-tongue. It can also discriminate at most five out of six different groups. The use of a low-level data fusion technique for the e-nose and e-tongue has enabled the six different kinds of *Orthosiphon stamineus* to be grouped separately. Six different groupings were observed, possibly due to different geographical origin, farming practices or postharvest processing among those brands. This fusion technique has improved the confidence level and discrimination performance by reducing uncertainties and allowing the e-nose and e-tongue to complement each other.

The performance of LDA to classify complex samples of *Orthosiphon stamineus* was also improved when the data from e-nose and e-tongue were fused together. The total percentage of variance for the first two PCs and the separation between the groups were also improved. Similar behavior of data clustering between two different types of sample preparations (dried and infusion) were also observed when using LDA. When the e-tongue was used separately, two sensors were not significant. However, after both e-nose and e-tongue were fused, the cross sensitivity effects among the sensors have been increased and all sensors were found to be significant and contributed towards the classification.

This investigation has proven that different sensor modalities can extract more information and hence by combining the modalities, the classification performance can be enhanced. This approach has enabled the evaluation and extraction of more information out complex samples which have high similarities between them.

In summary, by applying data fusion, the combined e-nose and e-tongue responses is analogous to the human sensing system as both interact and complement each other. Hence, this fusion method has strong potential to assist human panels in making decisions, for applications such as herbal quality assessments.

## Figures and Tables

**Table 1.** Samples used in the experiments and number of replicated measurements.

**Brands**	**Number of Samples**	Number of replicated e-nose measurements*	**Number of replicated e-tongue measurements**	**Colour of tea infusion**
Tropika	6	10	3	Light yellow
RainHill	6	10	3	Dark yellow
Polen	6	10	3	Very light yellow
Naturale	6	10	3	Light yellow
BioFeld	6	10	3	Very light yellow
Agro	6	10	3	Greenish

* Both for dried leaves and tea infusions

**Table 2.** Chalcogenide-based potentiometric electrodes used in e-tongue.

**Sensor Label**	**Description**
Fe^3+^	Ion-selective sensor for Iron ions
Cd^2+^	Ion-selective sensor for Cadmium ions
Cu^2+^	Ion-selective sensor for Copper ions
Hg^2+^	Ion-selective sensor for Mercury ions
Ti+	Ion-selective sensor for Titanium ions
S^2−^	Ion-selective sensor for Sulfur ions
Cr (VI)	Ion-selective sensor for Chromium ions

HI 5311	Reference probe using Ag/AgCl electrode

**Table 3.** E-nose parameter setting for *Orthosiphon stamineus* tea assessment.

**Sampling setting**	**Dried leaves**		**Tea infusion**	
**Cycle**	**Time(s)**	**Pump Speed**	**Time(s)**	**Pump Speed**
Baseline Purge	15	60 mL/min	10	120 mL/min
Sample Draw	20	60 mL/min	30	120 mL/min
Idle Time	3	-	3	-
Air Intake Purge	50	160/min	80	160 mL/min

**Table 4.** The amount of variance (%) of the first five principal components for three different experiments.

**Experiment**	**PC1**	**PC2**	**PC3**	**PC4**	**PC5**
Dried *Orthosiphon* leaves using e-nose	99.09	0.596	0.142	0.088	0.024
*Orthosiphon* tea infusions using e-nose	99.46	0.374	0.086	0.024	0.017
*Orthosiphon* tea infusions using e-tongue	85.12	6.095	4.220	1.906	1.526

**Table 5.** Wilks’ Lamda Test.

		**Before Fusion**		**After Fusion**	
**Modality**	**Sensor Label**	**Wilks' Lambda**	**F**	**Wilks' Lambda**	**F**
E-NOSE	SENSOR01	.209	22.672	.005	1,128.765
	SENSOR02	.200	23.988	.003	1,853.475
	SENSOR03	.290	14.707	.004	1,392.132
	SENSOR04	.161	31.174	.002	2,488.416
	SENSOR05	.366	10.393	.274	15.884
	SENSOR06	.211	22.449	.229	20.196
	SENSOR07	.045	128.200	.002	2,668.615
	SENSOR08	.180	27.277	.002	3,404.481
	SENSOR09	.022	268.335	.005	1,250.789
	SENSOR10	.015	391.406	.001	5,290.785
	SENSOR11	.036	159.070	.002	2,686.906
	SENSOR12	.169	29.496	.003	2,131.702
	SENSOR13	.037	154.903	.002	2,614.114
	SENSOR14	.038	150.154	.002	2,843.676
	SENSOR15	.028	209.879	.001	6,947.781
	SENSOR16	.024	239.119	.001	4,037.560
	SENSOR17	.020	286.910	.001	5,061.946
	SENSOR18	.120	44.176	.002	3,124.103
	SENSOR19	.239	19.096	.005	1,170.584
	SENSOR20	.266	16.516	.002	2,996.106
	SENSOR21	.031	186.642	.002	2,892.857
	SENSOR22	.019	307.061	.002	3,767.506
	SENSOR23	.298	14.143	.199	24.082
	SENSOR24	.163	30.855	.003	1,821.131
	SENSOR25	.054	105.628	.002	3,826.430
	SENSOR26	.243	18.692	.012	492.505
	SENSOR27	.522	5.499	.005	1,145.081
	SENSOR28	.228	20.373	.010	602.827
	SENSOR29	.031	188.591	.008	722.956
	SENSOR30	.045	126.357	.002	3,636.019
	SENSOR31	.390	9.402	.421	8.246
	SENSOR32	.408	8.693	.004	1,540.924
E-TONGUE	SENSOR01	.060	93.797	.000	16,946.764
	SENSOR02	.687	2.734	.520	5.547
	SENSOR03	.051	110.770	.194	25.003
	SENSOR04	.070	80.328	.000	30,371.417
	SENSOR05	.817	1.345	.771	1.782
	SENSOR06	.020	296.335	.000	57,563.900
	SENSOR07	.802	1.477	.645	3.305

## References

[1] Mendez MLR, Arrieta AA, Parra V, Bernal A, Vegas A, Villanueva S, Osuna RG, de Saja JA (2004). Fusion of three sensory modalities for the multimodal characterization of red wines. IEEE Sens. J. Vol.

[2] Guadarrama A, Fernández JA, Íñiguez M, Souto J, de Saja JA (2001). Discrimination of wine aroma using an array of conducting polymers sensors in conjunction with solid-phase micro-extraction (SPME) technique. Sens. Actuat. B: Chem.

[3] Schaller E, Zenhausern S, Zesiger T, Bosset JO, Escher F (2000). Use of preconcentration techniques applied to a MS-based “electronic nose”. Analysis.

[4] Gardner JW, Bartlett PN (1999). Electronic Noses: Principles and Applications.

[5] Lvova L, Legin A, Vlasov Y, Cha GS, Nam H (2003). Multicomponent analysis of Korean green tea by means of disposable all-solid-state potentiometric electronic tongue microsystem. Sens. Actuat. B:Chem.

[6] Hidayat W, Shakaff AYM, Ahmad MN, Adom AH (2010). Classification of agarwood oil using an electronic nose. Sensors.

[7] Bhattacharya N, Tudu B, Jana A, Ghosh D, Bandhopadhyaya R, Bhuyan M (2008). Preemptive identification of optimum fermentation time for black tea using electronic nose. Sens. Actuat. B: Chem.

[8] Dutta R, Kashwan KR, Bhuyan M, Hines EL, Gardner JW (2003). Electronic nose based tea quality standardization. Neural Netw.

[9] Legin AM, Rudnitskaya A, Yu G, Vlasov AV, Di Natale C, D'Amico A (1999). The features of the electronic tongue in comparison with the characteristics of the discrete ion-selective sensors. Sens. Actuat. B: Chem.

[10] Vlasov Y, Legin A, Rudnitskaya A, D’Amico A, Di Natale C (2000). Electronic tongue-new analytical tool for liquid analysis on the basis of non-specific sensors and methods of pattern recognition. Sens. Actuat. B: Chem.

[11] Toko K (2000). Taste sensor. Sens. Actuat. B: Chem.

[12] Persaud K, Dodd G (1982). Analysis of discrimination mechanisms in the mammalian olfactory system using a model nose. Nature.

[13] Delwiche JF (2004). The impact of perceptual interactions on perceived flavor. Food Qual. Prefer.

[14] Winquist F, Lundstrom I, Wide P (1999). The combination of an electronic tongue and an electronic nose. Sens. Actuat. B: Chem.

[15] Natale CD, Paolesse R, Macagnano A, Mantini A, D'Amico A, Legin A, Lvova L, Rudnitskaya A, Vlasov Y (2000). Electronic nose and electronic tongue integration for improved classification of clinical and food samples. Sens. Actuat. B: Chem.

[16] Boilot P, Hines EL, Gongora MA, Folland RS (2002). Electronic Noses inter-comparison, data fusion and sensor selection in discrimination of standard fruit solutions. Sens. Actuat. B: Chem.

[17] Winquist F, Holmin S, Krantz-Rulcker C, Wide P, Lundstrom I (2000). A hybrid electronic tongue. Anal. Chim. Acta.

[18] Sim CO, Ahmad MN, Ismail Z, Othman AR, Noor NA, Zaihidee EM (2003). Chemometric Classification of Herb—*Orthosiphon stamineus* according to its geographical origin using virtual chemical sensor based upon fast GC. Sensors.

[19] Legin A, Rudnitskaya A Chalcogenide Glass Electrodes.

[20] National Instruments NI USB-6008.

[21] Gardner JW, Boilot P, Hines EL (2005). Enhancing electronic nose performance by sensor selection using a new integer-based genetic algorithm. Sens. Actuat. B: Chem.

[22] Steinmetz V, Sevila F, Bellon-Maurel V (1999). A Methodology for sensor fusion design: Application to fruit quality assessment. J. Agric. Engng. Res.

[23] Berrueta LA, Alonso-Slaces RM, Heberger K (2007). Supervised pattern recognition in food analysis. J. Chrom: A.

[24] Tabachnick BG, Fidell LS (2007). Using Multivariate Statistics.

